# Monocytes and Macrophages in Alpha-1 Antitrypsin Deficiency

**DOI:** 10.2147/COPD.S276792

**Published:** 2020-12-03

**Authors:** Kylie B R Belchamber, Eloise M Walker, Robert A Stockley, Elizabeth Sapey

**Affiliations:** 1 Birmingham Acute Care Research Group, Institute of Inflammation and Ageing, University of Birmingham, Birmingham, UK; 2 NIHR Clinical Research Facility Birmingham, University Hospitals Birmingham NHS Foundation Trust, Birmingham, UK

**Keywords:** monocyte, macrophage, alpha-1 antitrypsin, alpha-1 antitrypsin deficiency

## Abstract

Alpha-1 antitrypsin deficiency (AATD) is a genetic condition characterised by low circulating levels of alpha-1 antitrypsin (AAT), a serine proteinase inhibitor. The most common deficiency variants are the S and Z mutations, which cause the accumulation of misfolded AAT in hepatocytes resulting in endoplasmic reticular stress and insufficient release of AAT into the circulation (<11μmol/L). This leads to liver disease, as well as an increased risk of emphysema due to unopposed proteolytic activity of neutrophil-derived serine proteinases in the lungs. AATD has been traditionally viewed as an inflammatory disorder caused directly by a proteinase-antiproteinase imbalance in the lung, but increasing evidence suggests that low AAT levels may affect other cellular functions. Recently, AAT polymers have been identified in both monocytes and macrophages from AATD patients and evidence is building that these cells may also play a role in the development of AATD lung disease. Alveolar macrophages are phagocytic cells that are important in the lung immune response but are also implicated in driving inflammation. This review explores the potential implications of monocyte and macrophage involvement in non-liver AAT synthesis and the pathophysiology of AATD lung disease.

## Introduction

Alpha-1 antitrypsin deficiency (AATD) is an autosomal co-dominant genetic condition that is characterised by low circulating levels of alpha-1 antitrypsin (AAT) protein, a serine proteinase inhibitor synthesised and secreted mainly by hepatocytes, but also by immune and other cells.^1^ AAT circulates in the blood and enters tissues including the lungs by diffusion, where it binds and inhibits proteinases including neutrophil elastase (NE), cathepsin G and proteinase 3, as well as potentially exhibiting anti-inflammatory properties.^2^ Low levels of AAT lead to an increased likelihood of developing emphysema, especially at an early age (30–40 years) and the accumulated protein in hepatocytes may lead to clinically overt liver disease.^1^ AAT is released as a single polypeptide chain that binds neutrophil elastase via a methionine residue at position 358 on the reactive centre loop of the protein. This then translocates to the opposite side of the protein and inserts the reactive loop into a β sheet, forming a covalently linked complex inactivating both the enzyme and inhibitor and is cleared from the circulation.^3^ In the lung, neutrophil elastase and other proteinases are released from immune cells, primarily neutrophils where they aid extravasation from the blood. AAT inactivates the neutrophil serine proteinases in order to limit any bystander lung damage and thus maintain a physiological proteinase-anti-proteinase balance important for maintaining lung integrity.^4^ AAT is an acute phase protein and plasma levels normally increase within hours of inflammation or infection adding extra protection. However, in AATD, little or no AAT is released as part of this acute phase response disturbing the physiological balance further.^5^

AATD is caused by a genetic mutation on the SERPINA1 gene that encodes the AAT protein and results in low circulating levels of the AAT protein in the ZZ variant (2–8µmol/L). An estimated 3.4 million people worldwide are affected; however, under diagnosis means many individuals remain undetected so this number is likely to be higher, highlighting the need for screening programmes in at risk individuals.^6^,^7^ There are over 120 reported mutations in this gene, with the most common being the S and Z mutations, which affect 1.4 million and 250,000 people worldwide, respectively.^8^ Both mutations result in misfolding of the protein, with the S variant resulting in a proportion of the AAT protein (~40%) being retained within hepatocytes, which reduces plasma levels but may not affect susceptibility to either liver or lung disease.^9–11^ The Z mutation occurs in over 95% of diagnosed AATD patients and causes greater misfolding and polymerisation of the protein. These polymers have no anti-neutrophil elastase activity and accumulate in the endoplasmic reticulum (ER) of cells. Eighty-five percent of the misfolded protein is retained in the hepatocytes and partially removed by ER degradation pathways, while the remaining 15% is secreted into the serum largely as monomers but still retains the potential to polymerise. The retention of polymers in the ER causes cell damage through ER stress, ER overload response, mitochondrial dysfunction and autophagy which damages cells and leads to liver disease with AAT protein overload, and lung disease due to circulating protein insufficiency.^1^,^12^

Although AAT is produced mainly by hepatocytes,^13^ it is also produced by lung epithelial cells,^14^ neutrophils,^15^,^16^ intestinal epithelial cells,^17^ and pancreatic islet cells.^18^ AATD polymers have been found in the lung^19^ (including the bronchoalveolar lavage), skin and kidneys,^20^ around capillaries and in epithelial cells.^21^ The lack of systemic AAT leads to a reduced ability to control serine proteinases in the lungs. This results over time in parenchymal lung destruction due to increased connective tissue breakdown, and the development of panlobular emphysema, especially in the presence of smoking.^22^ Z-AAT polymers also co-localise with neutrophils in the lung tissue, which increases the local proteinase release thereby contributing to further interstitial damage, as previously reviewed.^23^,^24^ Recently, AAT polymers have been identified in both monocytes^25^ and macrophages^26^ from AATD patients and evidence is building that these cells may also play a role in the development of AATD lung disease. The purpose of this review is to explore the potential implications of the local lung cells involved in non-liver AAT synthesis.

## Monocytes

Human monocytes account for 5–10% of circulating blood leukocytes. Monocytes are equipped with chemokine and adhesion receptors which mediate their migration from the blood to sites of infection or injury. Once migrated, activated monocytes release pro-inflammatory cytokines such as IL-1β, IL-6 and TNFα as well as other inflammatory mediators which induce the recruitment of further immune cell types, and moderates inflammation (for review see^27^,^28^). There are three main monocyte phenotypes – classical CD14^++^ CD16^−^, intermediate CD14^+^ CD16^+^ and non-classical CD14^−^, CD16+.^29^ Classical monocytes are primed for phagocytosis, migration and immune sensing. Intermediate monocytes express CCR5 and play a role in antigen presentation and cytokine secretion. Non-classical monocytes are involved in complement and Fcγ mediated phagocytosis and adhesion.^30^ However, these functions overlap and the spectrum of activity of these subsets is not exclusive.^28^ Once migrated to peripheral tissues, monocytes differentiate into macrophages or dendritic cells depending on the local milieu. This increases the phagocytic or antigen presentation capacity of the local environment as required, and aids in clearing the infectious agent/s and modulating the immune response. Monocytes are thus a vital component of the secondary immune response to infection.

## Monocytes and Alpha-1 Antitrypsin

Monocytes express the AAT gene^31^,^32^ at a level that is 200 times less than hepatocytes, indicating that they are a minor source of overall AAT but may contribute to the localised pool.^33^ Monocytes secrete AAT and also maintain this property when differentiated into macrophages.^31^ Alongside the main function of AAT to inhibit the activity of free neutrophil elastase locally, AAT also has some anti-inflammatory properties that may be enhanced by monocyte secretion locally. In monocytes, AAT inhibits lipopolysaccharide-stimulated release of pro-inflammatory cytokines TNFα and IL-1β, increases anti-inflammatory IL-10 release,^34^ and reduces CD14 expression^35^ via increased cAMP.^36^ Together this may prevent the over-activation of monocytes and other immune cells and would curtail the immune response, promoting the resolution of inflammation. This secondary role of AAT is independent of its anti-proteinase activity, as it occurs with both oxidised and heat-inactivated protein.^34^ This may important in the management of AATD, as it suggests that targeting the neutrophil elastase inhibitory function independently of AAT (for instance with chemical elastase inhibitors) may only partly restore normal homeostasis in the AATD lung, as the effect of AAT on other cell functions may be an important component for the development and treatment of lung disease.

## Monocytes in Alpha-1 Antitrypsin Deficiency

Monocyte subset analysis has shown that PiZZ patients have similar levels of HLA-DR^+^ monocyte subsets, but fewer non-classical HLA-DR^−^ cells. These latter cells protect the vascular endothelium during inflammation through the removal of damaged cells and secretion of anti-inflammatory mediators,^37^,^38^ and alteration in this subset variation has been suggested as a contributing factor to the early disease inflammation in AATD, although this is based on a single study of low numbers of patients that requires validation at least in vitro.^39^

**Figure 1:**
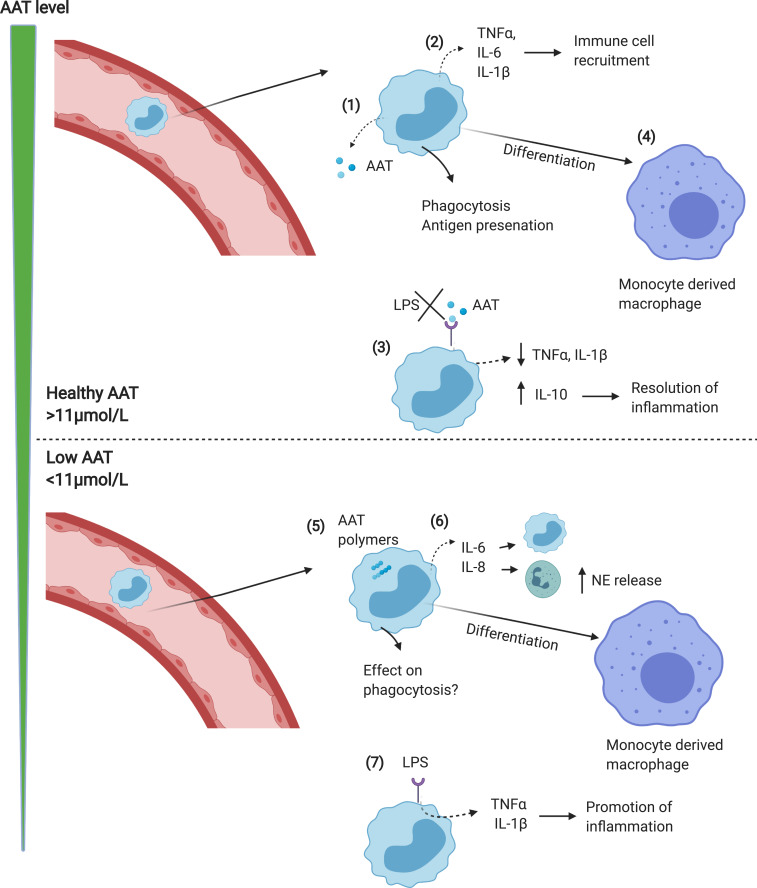
Monocyte function in normal vs AATD lungs. (1) In response to infection/inflammation, circulating monocytes enter the lungs where they release AAT which acts to neutralise NE. (2) Monocytes also release cytokines including TNFα, CXCL-8, LTB4 and IL-6 to recruit immune cells including neutrophils and monocytes. (3) AAT blocks LPS induced pro-inflammatory cytokine release, thus limiting inflammation. (4) Monocytes differentiate into monocyte-derived macrophages, adding to the alveolar macrophage pool. (5) In AATD, AAT polymers form in monocytes, leading to elevated release of pro-inflammatory cytokines including IL-6 and CXCL-8 which increases neutrophil recruitment (6), thus increasing NE levels and activity in the lungs. (7) Levels of AAT below the putative protective threshold (<11μmol/L) causes LPS induced pro-inflammatory cytokine release to increase, driving inflammation.

In response to neutrophil elastase, monocytes increase AAT mRNA; however, PiZZ monocytes (as with hepatocytes) secrete little AAT due to the accumulation of polymers within the cell.^32^ This leads to an “unfolded protein response” in the monocytes, with accumulation of AAT polymers within the ER, activation of NFĸB and increased expression of IL-6 and CXCL-8.^25^ Increased release of CXCL-8 would result in further recruitment of neutrophil to the lungs, increasing the elastase burden and further amplifying tissue degradation in the AAT deficient lung. IL-6 recruits’ further monocytes to the lungs perpetuating chronic neutrophil and monocyte induced inflammation (see Figure 1). This monocyte-driven loop may in some instances be the precipitating step in the initiation of a progressive destructive process in AATD especially in the 30% of never smokers who develop lung disease.^40^ The exact mechanism remains speculative but is worthy of further study. This also suggests that augmentation therapy in such individuals may work through both its anti-proteinase and anti-inflammatory effects.

## Macrophages

Alveolar macrophages line the alveoli and the airways of the lungs where they provide an important part of innate immunity, including phagocytosis of inhaled pathogens and releasing mediators to modulate the physiological immune response if required. The macrophage pool is long lived but also supplemented through the recruitment of monocytes, especially during acute and chronic episodes of inflammation, generating monocyte-derived macrophages with similar activity. Macrophages exhibit plasticity and are able to alter their phenotype and function in response to the local environmental requirements – from pro-inflammatory during infection, to anti-inflammatory during resolution of inflammation.^41^,^42^

## Macrophages and Alpha-1 Antitrypsin

Both peripheral lung macrophages isolated from bronchoalveolar lavage and monocyte-derived macrophages (MDM) transcribe the AAT gene, and secrete the protein.^32^ Monocyte differentiation to macrophages increases the level of gene expression 3-fold, though still 70 times less than hepatocytes, suggesting that macrophages contribute a greater component of local AAT in the lungs than monocytes. Furthermore, MDM differentiated into a pro-inflammatory phenotype using GM-CSF secrete even higher levels of AAT than MDM differentiated into an anti-inflammatory phenotype using M-CSF.^43^ LPS further increases gene expression of AAT in MDM, suggesting that exposure to gram negative bacteria in the lungs may drive AAT release from macrophages, which will contribute to the anti-proteinase activity during infection. How this relates to both the peripheral acute phase response and increased protein transudation from plasma during such episodes remains unknown.

AAT has several other effects on macrophages unrelated to its direct anti-proteinase activity. Neutrophil elastase binding to macrophages induces the release of LTB4,^44^ a major neutrophil chemoattractant in AATD,^45^,^46^ but this pathway is inhibited by AAT leading to reduced LTB4 release in vitro^44^ and in vivo.^47^ This reduces neutrophil recruitment to the airways, limiting the potential proteinase damage.

**Figure 2:**
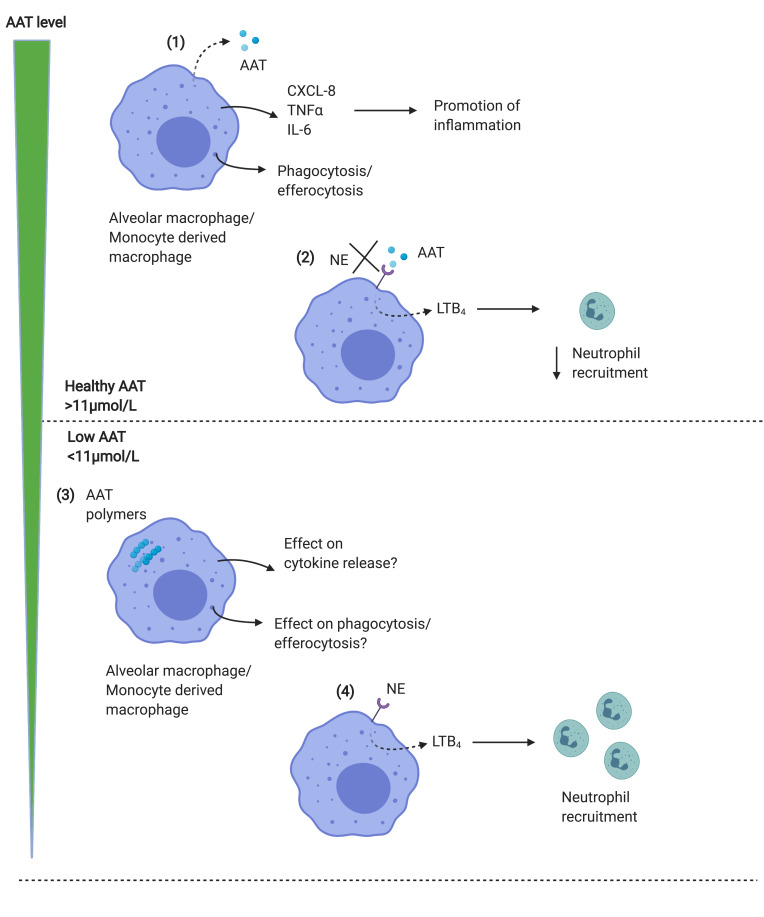
Macrophage function in normal vs AATD lungs. (1) Alveolar macrophages or MDM release AAT in response to inflammatory stimuli, together with proinflammatory cytokines including CXCL-8 and IL-6 which promote inflammation. Macrophages also phagocytose bacteria, and efferocytose apoptotic cells. (2) AAT acts to block the binding of neutrophil elastase to macrophage receptors, limiting the release of LTB4 resulting in reduced neutrophil recruitment to the airways, which limits inflammation. AAT is thus anti-inflammatory in the lungs. (3) In AATD, both MDM and alveolar macrophages contain AAT polymers, which may limit phagocytic function. (4) Levels of AAT below the putative protective threshold (<11μmol/L) results in the ability of NE to bind to receptors on macrophage surface, increasing LTB4 release and recruiting neutrophils, which further contribute to NE levels in the lungs, driving inflammation.

Exogenous AAT has also been shown to restore efferocytosis and phagocytosis by macrophages exposed to cigarette smoke, due to a reduction in shedding of CD206 and phosphatidylserine receptors.^48^ This suggests that the increased proteinase levels in the lungs of AATD patients may strip scavenger receptors from the macrophage surface in a similar manner to that shown in cystic fibrosis, where similar or even higher levels of proteinases are present,^49^ resulting in a reduced ability to bind bacteria as part of phagocytosis. However, this has yet to be studied in AATD. AAT is also able to suppress infection of MDM with *Mycobacterium abscessus* suggesting a potential to prevent bacterial infection in the lungs by acting directly on the macrophage.^50^ Further work is needed to elucidate the full effects of exogenous AAT on macrophage function and particularly the role in clearance of bacteria relevant to COPD (see Figure 2).

## Macrophages in Alpha-1 Antitrypsin Deficiency

Macrophages from PiZZ patients express normal levels of the AAT gene but release 10 times less AAT than normal^33^ resulting potentially in greater overall proteolytic activity associated with concomitant increased MMP14 expression.^51^ This is postulated to be due to the accumulation of AAT polymers within the cell, reducing the release of protein. However, a further study showed polymer accumulation did not occur in all PiZZ macrophages but only in 27% of cells observed in lung slices imaged by immunohistochemistry.^26^ Interestingly, the authors also found AAT polymers in lung slices from non-deficient smokers with and without COPD, but not in non-smokers which may reflect the effect of oxidants on polymerisation even with normal AAT. The authors also showed that the percentage of alveolar macrophage containing AAT polymers correlated with pack year smoking history, FEV_1_/FVC ratio, small airways dysfunction, CD8 T cell and neutrophil numbers in the alveolar walls, suggesting that polymer accumulation could be important in both AATD and non-deficient COPD. This observation is important as it is the first to show polymer accumulation within the macrophage, and it would be important to determine whether this relates to all macrophages within the slices and that the antibody used was specific to polymeric AAT.

In non-AATD COPD, macrophages are believed to be the key drivers of processes leading to disease initiation and progression.^52^ COPD macrophages are predominantly pro-inflammatory, display dysfunctional phagocytosis^53–55^ and efferocytosis,^56^ and have altered mitochondrial function.^57^ This likely contributes to the chronic colonisation of the respiratory tract, due to an inability to clear invading bacteria and apoptotic cells, despite the 20 fold increase in macrophage number within the lungs.^52^ Targeting macrophage function in COPD is a potential treatment strategy being explored. Whether this is also of importance in AATD macrophages requires exploration. The function of AATD macrophages is currently unknown, but the accumulation of polymers within the cell has implications for mechanisms such as phagocytosis and cytokine release.

## Macrophage-Neutrophil Crosstalk in AATD

The role of the neutrophil in AATD has been extensively reviewed;^23^,^24^ however, the impact of altered neutrophil function on macrophage biology has not been studied. Macrophages and neutrophils not only share similar functions, including phagocytosis and inflammatory mediator release, they also work in concert to coordinate the response to invading pathogens. This process is tightly regulated to modulate non-physiological damage to the host.

Macrophages recruit neutrophils to the lungs via the release of LTB_4_ and CXCL-8. Neutrophils are equally capable of recruiting monocytes to the lungs via the release of cathepsin G and azurocidin, which upregulates E-selectin and VCAM-1 on the vascular endothelium, promoting monocyte rolling, sticking and recruitment into the parenchyma.^58^,^59^ This results in increased numbers of both neutrophils and macrophages in the lungs during inflammation, especially to infectious organisms, to facilitate effective clearance of the invading organism.

Neutrophils are able to modify macrophage phenotype, and thereby modulate their function, through the release of reactive oxygen species, which activates macrophage AMPK, suppresses NFkB and induces an anti-inflammatory phenotype.^60^,^61^ This helps to limit the damage caused by these pro-inflammatory cells, and, on clearance of the invading organism induces the resolution of inflammation. During this, macrophages efferocytose apoptotic neutrophils in order to remove them from the tissue safely, and avoid excess proteinase release during necrosis. This process induces an anti-inflammatory macrophage phenotype which further promotes resolution of inflammation in the lungs once the initial infection is cleared. Efferocytosis occurs by binding of neutrophil apoptosis receptors, such as phosphatidylserine,^62^ annexin 1 and calreticulin^63^ to macrophage receptors including T cell immunoglobulin and mucin receptors (TIM) 1 and 4^64^ and integrins.^65^

In AATD, polymers have been shown to increased degranulation in airway neutrophil, resulting in increased apoptosis, possibly due to high levels of endoplasmic reticulum stress, and activation of the internal death pathways induced by polymers.^66^ This would lead to an increase in clearance requirements by alveolar macrophages, which may overwhelm the normal macrophage capacity, and may drive the macrophages into an anti-inflammatory phenotype, thus impairing the normal clearance of bacteria in the AATD lung. Clearly studies to examine this potential physiological failure should be undertaken as it may have a central role in AATD particularly during exacerbations when excess proteinase activity is generated. It is known that in non-AATD COPD, efferocytosis by alveolar macrophages is reduced,^56^ and since the inflammatory and neutrophilic response in AATD is enhanced this is likely to be a pathologically important pathway.^24^ Further, supplementation of macrophages in vitro with exogenous AAT restores cigarette smoke induced decrease in efferocytosis, suggesting AAT is protective to macrophages, although the mechanism is currently unknown.^48^

## Augmentation Therapy

Augmentation therapy is currently the only therapy specific for AATD approved in the US and Europe (though not in all countries including the UK). However, there are issues of cost-effectiveness and benefits to the patient which currently questions its use, particularly in the UK. Although augmentation can modulate (though not prevent) the progression of emphysema,^67–69^ likely due to the reduction in the effects of neutrophil elastase in the lungs and improvement of the serine proteinase:anti-proteinase imbalance, it cannot affect the formation of polymers within hepatocytes, monocytes or macrophages, and so these cells may remain in a pro-inflammatory state, and therefore continue causing damage through alternative pathways. However, studies are emerging which suggest potential beneficial effects of augmentation therapy on these cells themselves.

Augmentation restores AAT, which in turn reduces LTB4 production in airways due to the prevention of neutrophil elastase binding to macrophage surfaces.^44^ This will reduce neutrophil recruitment, and thereby reduce inflammation in the lungs. In mice, inhaled AAT restores cigarette smoke induced decrease in efferocytosis and also reduces neutrophil apoptosis,^48^ suggesting that AAT is cytoprotective although the mechanism is unclear. In human augmentation patients, MDMs isolated after an infusion of AAT are better able to control infection to *Mycobacterium intracellulare* due to greater phagosome-lysosome fusion and increased autophagosome formation/maturation.^70^ Whether this is also an effect with organisms more likely to be relevant to COPD and exacerbations needs to be confirmed however this suggests that augmentation therapy may potentially prevent bacterial exacerbation in AATD patients due to improved macrophage function, although this requires further and more relevant research. While augmentation therapy may slow progression of emphysema, the effect on lung function is less clear due to a lack of longitudinal studies, although observational studies suggest this to be the case.^71^ However, the impact of restoring lung AAT levels to normal on the whole immune response requires further research in order to determine whether this additional benefit aids in the treatment of this disease.

## Alternative Therapies

Alternative approaches to treating AATD are being developed, which target the polymeric AAT protein itself. These include chemical chaperones, which reverse cellular mislocalisation and misfolding of the protein, such as 4-phenylbutyric acid (PBA). PBA which has been shown to increase the secretion of functional Z-AAT in human skin fibroblasts expressing Z-AAT, and produced a 20–50% increase in serum AAT level in PiZZ mice.^72^ In human trials, PBA improves release of AAT from hepatocytes, and increased serum levels, but was associated with severe side effects.^73^ Further trials on the benefits of these chaperones on cellular AAT polymers, in hepatocytes and immune cells may show benefit in improving macrophage function.

Alternatively, small molecule correctors facilitate proper folding of the AAT protein within affected cells, and thus may increase systemic (via hepatocytes) and local release of AAT (by macrophage/monocytes). These include small peptides that fit into the open beta sheet of abnormal AAT, to block insertion of the inhibitory site loop of one AAT into the sheet of the next,^74^ thus preventing polymerisation. However, although this may prevent polymerisation it also inactivates the protein as an inhibitor and thus the main benefit may relate to protection of the liver and not the lung. To date, there is no published data on the effectiveness of these drugs in humans.^75^

## Conclusion

AATD is typically considered a disease driven by the proteolytic nature of neutrophil enzymes, but evidence is increasing that other cells of the innate and secondary immune system are also implicated. Fully understanding the interactive role and function of monocytes and macrophages in AATD may help explain the partial response to augmentation therapy and identify important alternative targets for treatment, and determine whether drugs that remove polymers from within the cells is essential for effective disease treatment.
